# Enhanced Reactivity of Aluminum Complexes Containing P-Bridged Biphenolate Ligands in Ring-Opening Polymerization Catalysis

**DOI:** 10.3389/fchem.2018.00607

**Published:** 2018-12-13

**Authors:** Xue-Ru Zou, Yu-Ning Chang, Kuo-Wei Huang, Lan-Chang Liang

**Affiliations:** ^1^Department of Chemistry, National Sun Yat-sen University, Kaohsiung, Taiwan; ^2^KAUST Catalysis Center and Division of Physical Sciences and Engineering, King Abdullah University of Science and Technology, Thuwal, Saudi Arabia; ^3^Department of Medicinal and Applied Chemistry, Kaohsiung Medical University, Kaohsiung, Taiwan

**Keywords:** aluminum, biphenolate, ring-opening polymerization, lactone, lactide, catalyst

## Abstract

Aluminum complexes containing [RP(O)(2-O-3,5-*t*Bu_2_C_6_H_2_)_2_]^2−^ [R = *t*Bu (**3a**), Ph (**3b**)] have been synthesized, structurally characterized, and their reactivity studied in comparison with those of their [RP(2-O-3,5-*t*Bu_2_C_6_H_2_)_2_]^2−^ [R = *t*Bu (**2a**), Ph (**2b**)] analogs. Treating AlMe_3_ with one equiv of H_2_[**3a-b**] in THF at 0°C affords quantitatively [**3a-b**]AlMe, subsequent reactions of which with benzyl alcohol in THF at 25°C generate {[**3a-b**]Al(μ_2_-OCH_2_Ph)}_2_. The methyl [**3a-b**]AlMe and the benzyloxide {[**3a-b**]Al(μ_2_-OCH_2_Ph)}_2_ are all active for catalytic ring-opening polymerization (ROP) of ε-caprolactone and *rac*-lactide (*rac*-LA). Controlled experiments reveal that {[**3a**]Al(μ_2_-OCH_2_Ph)}_2_ is competent in living polymerization. Kinetic studies indicate that [**3a**]AlMe, in the presence of benzyl alcohol, catalyzes ROP of *rac*-LA at a rate faster than [**3b**]AlMe and [**2a**]AlMe(THF) by a factor of 1.8 and 23.6, respectively, highlighting the profound reactivity enhancement in ROP catalysis by varying the P-substituents of these biphenolate complexes of aluminum.

## Introduction

**Figure 1:**
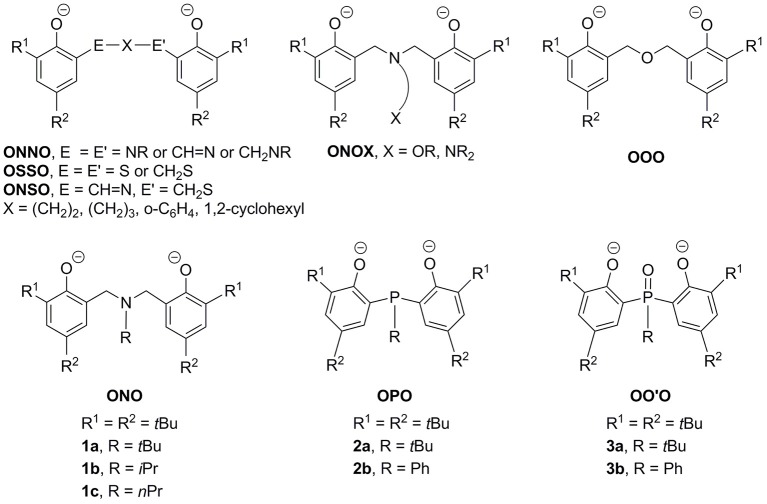
Representative examples of chelating biphenolate ligands.

The search for efficient catalyst precursors or initiators for catalytic ring-opening polymerization (ROP) of cyclic esters continues to constitute an active area of exploratory chemistry (Kamber et al., 2007; Thomas, 2010; Hillmyer and Tolman, 2014; Sarazin and Carpentier, 2015). In this regard, metal complexes containing chelating biphenolate ligands have attracted significant attention. These complexes are intriguing as their catalytic activities are finely tunable by judiciously varying the peripheral substituents on the two phenolate rings and/or the bridge in between. While most studies concentrate on metal complexes of tetradentate biphenolate ligands such as ONNO (Ovitt and Coates, 2000; Zhong et al., 2002; Hormnirun et al., 2006; Zelikoff et al., 2009; Chen et al., 2012; Gao et al., 2015; Jones et al., 2015; Kirk et al., 2016; MacDonald et al., 2016; McKeown et al., 2016; Robert et al., 2017; Pang et al., 2018), OSSO (Buffet and Okuda, 2011; Buffet et al., 2011), ONSO (Stopper et al., 2012), and ONOX (X = OR, NR_2_) (Alcazar-Roman et al., 2003; Gendler et al., 2006; Tang and Gibson, 2007; Phomphrai et al., 2010; Wichmann et al., 2012) as exemplified in Figure 1, parallel research centered upon tridentate counterparts is relatively rare (Chmura et al., 2006; Chang and Liang, 2007; Hsu and Liang, 2010; Liang et al., 2011, 2013a,b,c,d,e; Huang et al., 2013; Klitzke et al., 2014a,b; Chang et al., 2016).

It has been documented that complexes of tridentate OOO (Huang et al., 2013), ONO (**1a-c**) (Liang et al., 2013a,b,c,d,e), and OPO (**2a-b**) (Chang and Liang, 2007; Hsu and Liang, 2010; Liang et al., 2011; Chang et al., 2016) are active catalyst precursors for ROP of ε-caprolactone (ε-CL) or lactides (LAs). Studies on complexes of **1** and **2** have revealed that substituents at the pnictogen donor have decisive impacts on ROP catalysis if living polymerization is the goal. Of note are aluminum complexes of **2a** that polymerize ε-CL and *rac*-LA in a living manner to produce well-defined poly(ε-caprolactone) (PCL) and poly(*rac*-lactide) (PLA), respectively (Chang et al., 2016). In contrast, analogous complexes of **1a** give instead low molecular weight oligo(ε-caprolactone) or PCL with a somewhat broad molecular weight distribution (Liang et al., 2013b). Constitutionally, complexes of **1a** and **2a** are much alike as both are pnictogen biphenolate derivatives carrying a pnictogen-bound *tert*-butyl group. Having an extra benzylic methylene moiety in each arm, however, the former ligand, upon complexation, forms 6-membered chelating rings that are therefore less rigid than the 5-membered chelating rings derived from the latter. In an effort to better understand the effects of chelating ring size (Lee et al., 2017) and ligand rigidity (Liang et al., 2003a,b, 2005a,b, 2006; Huang and Liang, 2004; MacLachlan and Fryzuk, 2005; Liang, 2006; MacLachlan et al., 2007; Hung et al., 2014) on ROP catalysis, we turn our attention to the oxidative forms of **2** that would persist with the same rigidity but enlarge the chelating rings to be 6-membered. Such biphenolate phosphinoxide derivatives OO'O are distinguished from the ether-bridged OOO types that are intrinsically more flexible in ligand backbone and lack of the opportunities of changing substituents at the bridge donor. Note that complexes of OO'O types of ligands are relatively undeveloped (Tanke et al., 1991; Siefert et al., 2000; Paine et al., 2004; He et al., 2008; Zhang et al., 2013; Taniyama et al., 2014). In this contribution, we aim to demonstrate the syntheses of the first examples of OO'O complexes of aluminum and their enhanced catalytic activities in comparison with those of **2** with respect to ROP of ε-CL and *rac*-LA.

## Results and Discussion

### Ligand Synthesis

The protio ligand precursor H_2_[**3b**] is known (Siefert et al., 2000). Its *tert*-butyl analog H_2_[**3a**] can be readily prepared as an off-white solid in high yield from oxidation of H_2_[**2a**] with hydrogen peroxide in THF under ambient conditions. Its solution NMR data are consistent with a structure having time-averaged *C*s symmetry. The diagnostic signals of this compound involve the downfield shift of its phosphorus atom at 65 ppm in comparison with that of H_2_[**2a**] at −60 ppm (Hsu and Liang, 2010) and the singlet resonance of its hydroxy protons at 12.19 ppm in comparison with the doublet resonance of those in H_2_[**2a**] at 7.61 ppm with *J*_HP_ = 12 Hz (Hsu and Liang, 2010). The lack of OH⋯ P internuclear coupling and the downfield shift of the hydroxy protons in H_2_[**3a**] are apparently a consequence of the 6-membered OH⋯ O = P hydrogen bonding. Such intramolecular hydrogen bonding is also confirmed by the solid state structure of H_2_[**3a**] established by an X-ray diffraction study (Figure S1, Table S1).

### Synthesis and Characterization of Aluminum Complexes

**Figure 2:**
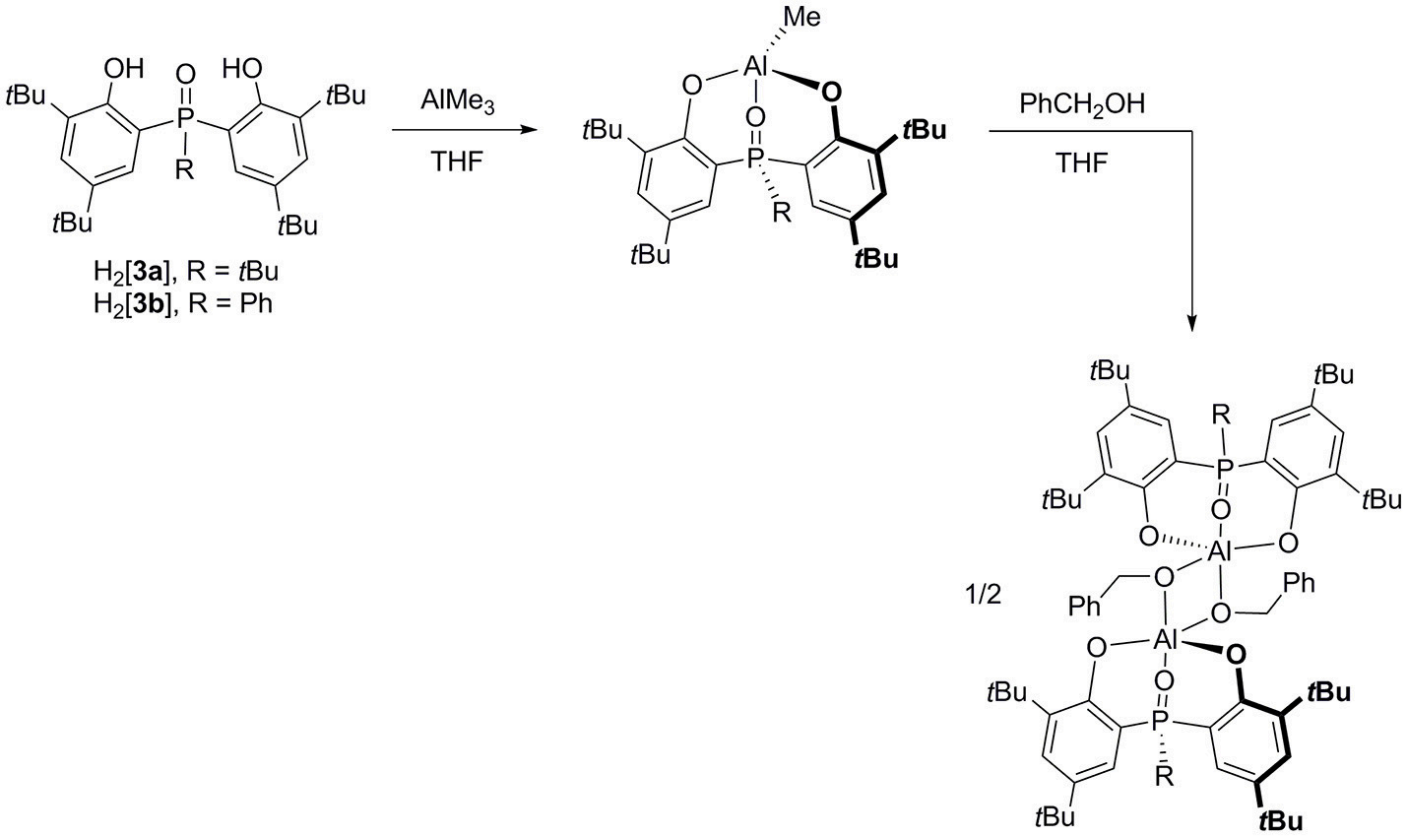
Synthesis of biphenolate phosphinoxide complexes of aluminum.

Protonolysis of AlMe_3_ with one equiv of H_2_[**3a-b**] in THF at 0°C yields nearly quantitative [**3a-b**]AlMe (Figure 2). Interestingly, these methyl complexes are not THF-bound as evidenced by their ^1^H NMR spectra. This result is reminiscent of 4-coordinate [**1a–c**]AlMe (Liang et al., 2013b) but in contrast to 5-coordinate [**2a-b**]AlMe(THF) (Chang et al., 2016), ascribable to the hardness similarity of O (from phosphinoxide in **3**) to N (from **1**) rather than P (from **2**) in consideration of the distinct preferences of these hard and soft donors to bind to a hard aluminum (Fryzuk et al., 1996, 1998; Liang et al., 2004, 2010; Lee and Liang, 2005, 2009; Su and Liang, 2018). As a result, the solution structures of [**3a-b**]AlMe and [**1a–c**]AlMe should be much alike. Subsequent reactions of either isolated or *in situ* prepared [**3a-b**]AlMe with one equiv of benzyl alcohol in THF at 25°C afford {[**3a-b**]Al(μ_2_-OCH_2_Ph)}_2_ as colorless crystals.

The solution NMR data of [**3a-b**]AlMe and {[**3a-b**]Al(μ_2_-OCH_2_Ph)}_2_ are indicative of a mirror plane symmetry that makes the two phenolate rings in **3** chemically equivalent as evidenced by the observation of two distinct singlet resonances for arylated *tert*-butyl groups in the ^1^H NMR spectra. The methylene groups in the benzyloxide ligands of {[**3a-b**]Al(μ_2_-OCH_2_Ph)}_2_ exhibit a singlet resonance in the ^1^H NMR spectra at ca. 5.7 ppm, a chemical shift that is similar to that found for {[**2a-b**]Al(μ_2_-OCH_2_Ph)}_2_ (Chang et al., 2016). A ^1^H NMR NOE difference experiment of {[**3a**]Al(μ_2_-OCH_2_Ph)}_2_ was conducted with selective irradiation on the methylene groups, resulting in NOE enhancements of 5.14% for the arylated *tert*-butyl groups ortho to the phenolate oxygen atoms and 3.21% for the P-bound *tert*-butyl group. Note that these *tert*-butyl groups are spatially far away from each other due to the inherent geometry of the facially tridentate **3a**. The concurrent NOE enhancements on these *tert*-butyl groups thus strongly implicate a dimeric structure of {[**3a**]Al(μ_2_-OCH_2_Ph)}_2_ in solution. Consistent with the oxidized characteristics of the phosphorus atom in phosphinoxide derivatives, the ^31^P chemical shifts of [**3a-b**]AlMe and {[**3a-b**]Al(μ_2_-OCH_2_Ph)}_2_ are significantly downfield shifted from those of their corresponding analogs of **2** (Chang et al., 2016).

**Figure 3:**
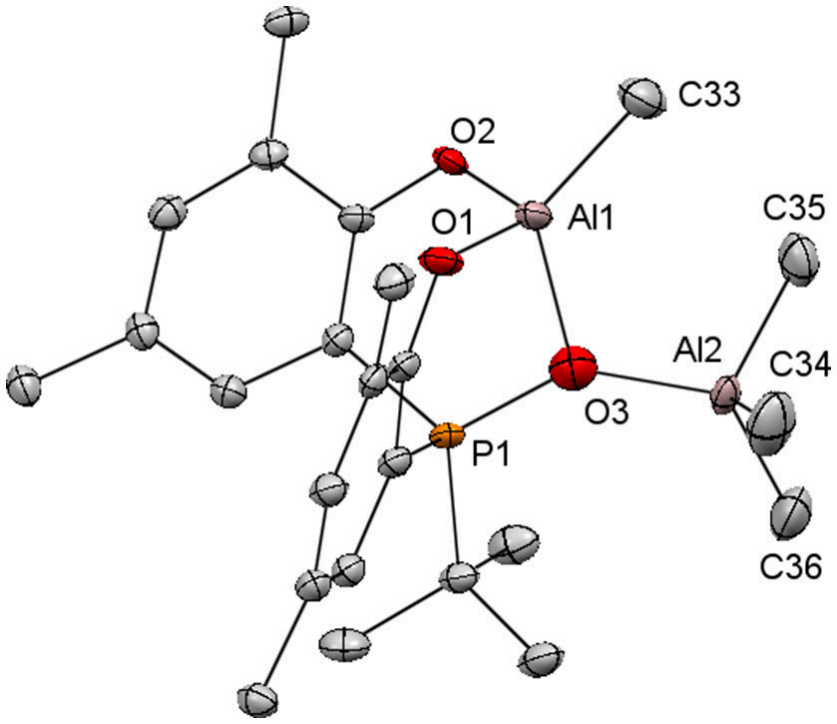
Molecular structure of [**3a**]AlMe•AlMe_3_ with thermal ellipsoids drawn at the 35% probability level. All hydrogen atoms and the methyl groups in arylated *tert*-butyls are omitted for clarity. Selected bond distances (Å) and angles (deg): Al(1)-O(2) 1.749(3), Al(1)-O(1) 1.764(3), Al(1)-C(33) 1.933(5), Al(1)-O(3) 1.934(4), Al(2)-O(3) 1.753(4), Al(2)-C(34) 1.844(6), Al(2)-C(36) 1.872(6), Al(2)-C(35) 1.872(5), O(3)-P(1) 1.623(4), O(2)-Al(1)-O(1) 110.75(17), O(2)-Al(1)-C(33) 111.5(2), O(1)-Al(1)-C(33) 108.6(2), O(2)-Al(1)-O(3) 98.48(17), O(1)-Al(1)-O(3) 99.39(16), C(33)-Al(1)-O(3) 127.0(2), O(3)-Al(2)-C(34) 107.6(3), O(3)-Al(2)-C(36) 121.0(2), C(34)-Al(2)-C(36) 107.5(3), O(3)-Al(2)-C(35) 107.2(2), C(34)-Al(2)-C(35) 111.6(3), C(36)-Al(2)-C(35) 101.8(3), P(1)-O(3)-Al(2) 141.8(2), P(1)-O(3)-Al(1) 103.2(2), Al(2)-O(3)-Al(1) 112.5(2).

An attempt to characterize [**3a**]AlMe by X-ray crystallography led instead to the structure of [**3a**]Al_2_Me_4_ that is an O-bound AlMe_3_ adduct of [**3a**]AlMe (Figure 3). We attribute this unexpected result to unintentional imbalance of reaction stoichiometry that gives a trace amount of highly crystalline [**3a**]AlMe•AlMe_3_. Following this lead, we attempted the reactions of [**3a**]AlMe with one equiv of AlMe_3_ or H_2_[**3a**] with two equiv of AlMe_3_. Unfortunately, these reactions result ultimately in a mixture of equal molar [**3a**]AlMe and AlMe_3_ as evidenced by ^1^H and ^31^P{^1^H} NMR spectra of reaction aliquots. Subsequent attempts to crystallographically characterize [**3a**]AlMe have thus far been unsuccessful. Nevertheless, the structure of [**3a**]AlMe•AlMe_3_ confirms the 4-coordinate nature for the aluminum center of the [**3a**]AlMe moiety that has a distorted tetrahedral coordination core similar to [**1a–c**]AlMe (Liang et al., 2013b). The bond distances and angles of [**3a**]AlMe•AlMe_3_ are unexceptional.

**Figure 4:**
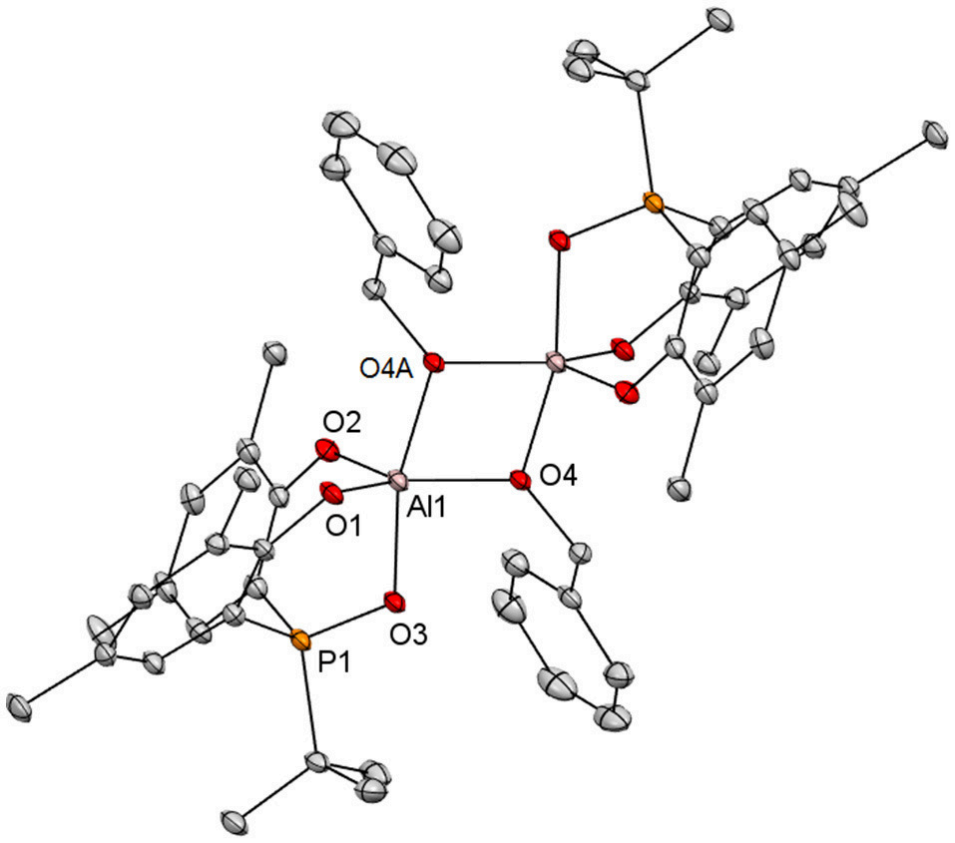
Molecular structure of {[**3a**]Al(μ_2_-OCH_2_Ph)}_2_ with thermal ellipsoids drawn at the 35% probability level. All hydrogen atoms and the methyl groups in arylated *tert*-butyls are omitted for clarity. Selected bond distances (Å) and angles (deg): Al(1)-O(2) 1.7766(18), Al(1)-O(1) 1.7890(18), Al(1)-O(4) 1.8372(17), Al(1)-O(3) 1.8414(17), Al(1)-O(4A) 1.8726(17), Al(1)-Al(1A) 2.9360(14), O(3)-P(1) 1.5274(16), O(2)-Al(1)-O(1) 109.46(9), O(2)-Al(1)-O(4) 117.94(9), O(1)-Al(1)-O(4) 132.10(9), O(2)-Al(1)-O(3) 94.74(8), O(1)-Al(1)-O(3) 92.52(8), O(4)-Al(1)-O(3) 90.14(8), O(2)-Al(1)-O(4A) 95.13(8), O(1)-Al(1)-O(4A) 94.67(8), O(4)-Al(1)-O(4A) 75.37(8), O(3)-Al(1)-O(4A) 165.10(8), Al(1)-O(4)-Al(1A) 104.63(8), P(1)-O(3)-Al(1) 114.84(10).

Colorless crystals of {[**3a**]Al(μ_2_-OCH_2_Ph)}_2_ suitable for X-ray diffraction analysis were grown by layering pentane on top of a concentrated THF solution at −35°C. Figure 4 depicts its solid state structure. Consistent with the NOE study, this complex is a dimer, composed of two [**3a**]Al(OCH_2_Ph) units bridged with the benzyloxide ligands. With the coordination of the facially tridentate **3a**, the aluminum atoms in {[**3a**]Al(μ_2_-OCH_2_Ph)}_2_ are therefore 5-coordinate. Its coordination geometry is best described as distorted trigonal bipyramidal, having the phosphinoxide donor and one of the bridging benzyloxide ligands disposed at the axial positions [O(3)-Al(1)-O(4A) = 165.10(8)°]. The axial Al-OCH_2_Ph bond distances of 1.8726 (17) Å are longer than those disposed equatorially [1.8372 (17) Å]. This indicates that the equatorial benzyloxide ligands are more anionic in nature whereas those at the axial positions are more dative. Consistent with this result, [**2a**]AlMe(THF), though constitutionally different, holds a methyl ligand equatorially, and a THF axially (Chang et al., 2016).

The P-bound *tert*-butyl groups in [**2a**]AlMe(THF) and {[**2a**]Al(μ_2_-OCH_2_Ph)}_2_ are known to sterically repulse their equatorial methyl and benzyloxide ligands away from the ideal positions with the P-Al-C and P-Al-O angles of 105.51(12)° and 107.25(9)°, respectively (Chang et al., 2016). Such steric repulsion is apparently eased by moving the *tert*-butyl-bound phosphorus atom to the β position in {[**3a**]Al(μ_2_-OCH_2_Ph)}_2_ as evidenced by the O(3)-Al(1)-O(4) angle of 90.14(8)°. With the incorporation of the rigid *o*-phenylene backbone and the 6-membered chelating rings in {[**3a**]Al(μ_2_-OCH_2_Ph)}_2_, the O(1)-Al(1)-O(3) [92.52(8)°] and O(2)-Al(1)-O(3) [94.74(8)°] angles are notably wider than the corresponding angles (80.35(9)° and 84.17(9)°) in the 5-membered chelating rings of {[**2a**]Al(μ_2_-OCH_2_Ph)}_2_ (Chang et al., 2016). As such, the *tert*-butyl groups ortho to the phenolate oxygen atoms in the former are spatially closer to the axial benzyloxide ligands than those in the latter. This should in principle encourage dissociation of the datively bonded benzyloxide ligands and formation of transient monomeric [**3a**]Al(OCH_2_Ph) for subsequent substrate coordination. The similarity of Al(1)-O(4A) distance [1.8726(17) Å] to that in {[**2a**]Al(μ_2_-OCH_2_Ph)}_2_ [1.878(2) Å] (Chang et al., 2016) implicates surprisingly little difference in trans influence invoked by P = O and P for axial benzyloxide ligand dissociation from these aluminum complexes.

### Catalytic Ring-Opening Polymerization

Similar to aluminum derivatives of **1** (Liang et al., 2013b) and **2** (Chang et al., 2016), complexes [**3a-b**]AlMe and {[**3a-b**]Al(μ_2_-OCH_2_Ph)}_2_ are all active for catalytic ROP of ε-CL and *rac*-LA. To establish parallel comparison on reactivity of these pnictogen derivatives, the catalysis of [**3**]AlMe and {[**3**]Al(μ_2_-OCH_2_Ph)}_2_ was examined under conditions identical to those employed for [**1**]AlMe (Liang et al., 2013b), [**2**]AlMe(THF) (Chang et al., 2016), and {[**2**]Al(μ_2_-OCH_2_Ph)}_2_ (Chang et al., 2016). To simplify tabulated discussion and to make consistency with other mononuclear species, the benzyloxide complexes are presented as a monomer. Table 1 summarizes their catalytic activities with ε-CL. In the presence of one equiv of benzyl alcohol, [**3a**]AlMe reacts slightly faster than [**3b**]AlMe with 100 equiv of ε-CL under the conditions employed (entry 1 vs. 3) though both reactions complete in 2 h (entries 2 and 4). The observed number averaged molecular weights (*M*n's), however, are generally smaller than those expected. Studies on *M*n's of these PCLs by ^1^H NMR spectroscopy also give similar results (entry 2, 5.1 kg/mol; entry 4, 9.5 kg/mol). Both [**3a**]AlMe and [**3b**]AlMe are more reactive than [**2a**]AlMe(THF) and [**2b**]AlMe(THF) (entries 1 and 3 vs. 5 and 6) due apparently to the discrepancy of **3** and **2** that invokes THF coordination and retards ROP. Though [**1a**]AlMe, [**2a**]AlMe(THF), and [**3a**]AlMe are all *tert*-butylated at their pnictogen atom, the polydispersity indexes (PDIs) of PCLs produced from [**2a**]AlMe(THF) and [**3a**]AlMe are smaller than those from [**1a**]AlMe (entries 1–2 and 5 vs. 7–8). All in all, [**3a**]AlMe is therefore a superior catalyst precursor to [**2a**]AlMe(THF) that in turn outperforms [**1a**]AlMe in this catalysis. These results underscore the decisive role that the biphenolate bridges play in ROP catalysis.

**Table 1:** ROP of ε-CL by catalytic [**3a-b**]AlMe and [**3a-b**]Al(OCH_2_Ph)^a^.

**Entry**	**Cat**	**[cat]_**0**_/[I]_**0**_/[ε-CL]_**0**_**	**Time (h)**	**conv (%)^**b**^**	***M*n (calcd, kg/mol)^**c**^**	***M*n (exp, kg/mol)^**d**^^,^^**e**^**	**PDI^**d**^**
1	[**3a**]AlMe	1/1/100	1	72	8.3	3.4	1.14
2	[**3a**]AlMe	1/1/100	2	>99	11.5	5.2	1.15
3	[**3b**]AlMe	1/1/100	1	60	7.0	4.3	1.15
4	[**3b**]AlMe	1/1/100	2	>99	11.5	10.1	1.49
5^f^	[**2a**]AlMe(THF)	1/1/100	1	45	5.2	3.4	1.08
6^f^	[**2b**]AlMe(THF)	1/1/100	1	32	3.8	3.1	1.14
7	[**1a**]AlMe	1/1/100	1	35	4.1	3.6	1.36
8^f^	[**1a**]AlMe	1/1/100	3	>99	11.5	10.6	1.38
9	[**3a**]Al(OCH_2_Ph)	1/0/100	2	>99	11.5	5.4	1.11
10^g^	[**3a**]Al(OCH_2_Ph)	1/0/100	2	82	9.5	4.8	1.15
11	[**3b**]Al(OCH_2_Ph)	1/0/100	2	>99	11.5	10.6	1.29
12	[**3a**]Al(OCH_2_Ph)	1/0/200	3	>99	22.9	10.8	1.07
13	[**3a**]Al(OCH_2_Ph)	1/0/300	4	>99	34.4	14.9	1.07
14	[**3a**]Al(OCH_2_Ph)	1/0/400	4	>99	45.8	22.1	1.07

a *Unless otherwise noted, all reactions were conducted in toluene (2.24 mL total) at 70°C with benzyl alcohol being the initiator, [cat]_0_ = 8.3 mM*.

b *Determined by ^1^H NMR analysis*.

c *Calculated from {fw of ε-CL × ([ε-CL]_0_/([cat]_0_[I]_0_)) × conversion} + fw of initiator, assuming one propagating chain per aluminum atom*.

d *Measured by GPC in THF, calibrated with polystyrene standards*.

e *Multiplied by a corrected factor of 0.56 (Save et al., 2002)*.

f *Data selected from Chang et al. (2016)*.

g *Reaction run in THF*.

Similar to that generated *in situ*, [**3a**]Al(OCH_2_Ph) finishes polymerization of 100 equiv of ε-CL in toluene at 70°C in 2 h, producing quantitatively PCL having comparable *M*n and PDI (entry 9 vs. 2). End group analysis by ^1^H NMR spectroscopy reveals a benzyl ester functionality, implicating that this ROP proceeds with a coordination-insertion mechanism that involves ε-CL coordination to the transient monomeric [**3a**]Al(OCH_2_Ph) (vide supra) followed by insertion of this ε-CL into the Al-OCH_2_Ph bond, allowing ε-CL to ring-open by cleaving its acyl-oxygen bond for chain propagation. Consistent with this rationale, the same reaction conducted in THF proceeds relatively slower (entry 10). Nevertheless, the PCL thus produced has a satisfactorily small PDI, indicating that the interfering THF coordination is reversible and does not much induce undesirable side reactions.

**Figure 5:**
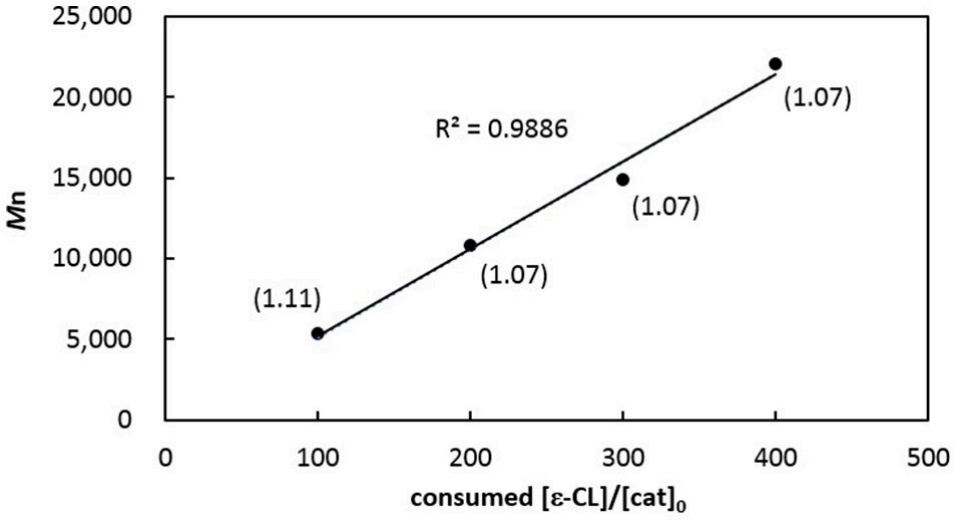
Linear plot of corrected *M*n of PCLs vs. monomers consumed to [**3a**]Al(OCH_2_Ph) ratios (entries 9 and 12–14 in Table 1). Numbers shown in parentheses indicate their corresponding PDIs.

In contrast, PCL produced from catalytic [**3b**]Al(OCH_2_Ph) has a relatively larger PDI (entry 11), reminiscent of that acquired from [**3b**]AlMe as compared with [**3a**]AlMe (entries 4 vs. 2). Complexes [**3a**]AlMe and [**3a**]Al(OCH_2_Ph) thus outperform [**3b**]AlMe and [**3b**]Al(OCH_2_Ph) in this catalysis. Interestingly, [**3a**]Al(OCH_2_Ph) polymerizes ε-CL in a living fashion. The PCLs thus produced (entries 9 and 12–14) have *M*n's linearly proportional to the consumed monomer-to-catalyst ratios (Figure 5) while maintaining consistently small PDIs.

**Figure 6:**
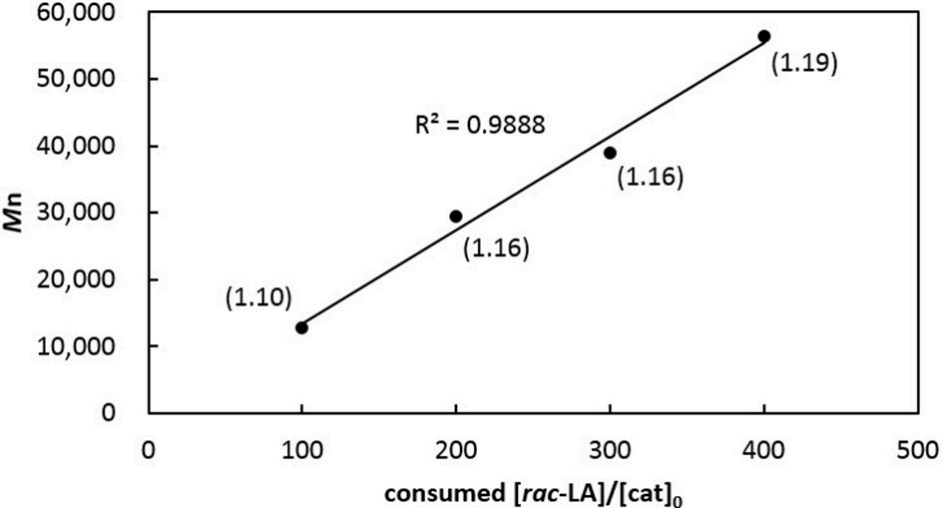
Linear plot of corrected *M*n of PLAs vs. monomers consumed to [**3a**]Al(OCH_2_Ph) ratios (entries 5–8 in Table 2). Numbers shown in parentheses indicate their corresponding PDIs.

Table 2 summarizes ROP results with respect to *rac*-LA. In the presence of one equiv of benzyl alcohol, [**3a**]AlMe and [**3b**]AlMe complete polymerization of 100 equiv of *rac*-LA in toluene at 70°C in 6 h, producing PLAs quantitatively (entries 1–2). The former complex is again a superior catalyst precursor to the latter in view of the smaller PDI derived. These reactions are faster than those by catalytic [**2a**]AlMe(THF) and [**2b**]AlMe(THF) (entries 3–4) (Chang et al., 2016). Interestingly, [**3a**]Al(OCH_2_Ph) is also competent in living ROP of *rac*-LA (entries 5–8), affording PLAs having *M*n's directly proportional to the consumed monomer-to-catalyst ratios (Figure 6) while keeping their PDIs consistently small. In contrast, PLA produced by catalytic [**3b**]Al(OCH_2_Ph) has a relatively larger PDI (entry 9). A reaction run in THF is again slow but does not change PDI much (entry 10). Catalysis run at room temperature results in slow reaction and low conversion (entry 11). In the presence of one equiv of poly(ethylene glycol) methyl ether (*M*n 2000, denoted MePEG2000 in Table 2), [**3a**]AlMe polymerizes *rac*-LA to give PEG-*b*-PLA copolymers with satisfactorily small PDIs (entries 12–13).

**Table 2:** ROP of *rac*-LA by catalytic [**3a-b**]AlMe and [**3a-b**]Al(OCH_2_Ph)^a^.

**Entry**	**Cat**	**[cat]_**0**_/[I]_**0**_/[*rac*-LA]_**0**_**	**Time (h)**	**Conv (%)^**b**^**	***M*n (calcd, kg/mol)^**c**^**	***M*n (exp, kg/mol)^**d**^^,^^**e**^**	**PDI^**d**^**
1	[**3a**]AlMe	1/1/100	6	>99	14.5	12.0	1.09
2	[**3b**]AlMe	1/1/100	6	>99	14.5	10.5	1.34
3^f^	[**2a**]AlMe(THF)	1/1/100	7	40	5.9	3.0	1.08
4^f^	[**2b**]AlMe(THF)	1/1/100	7	34	5.0	2.3	1.10
5	[**3a**]Al(OCH_2_Ph)	1/0/100	6	>99	14.5	12.8	1.10
6	[**3a**]Al(OCH_2_Ph)	1/0/200	6	>99	28.9	29.4	1.16
7	[**3a**]Al(OCH_2_Ph)	1/0/300	6	>99	43.3	39.0	1.16
8	[**3a**]Al(OCH_2_Ph)	1/0/400	6	>99	57.8	56.5	1.19
9	[**3b**]Al(OCH_2_Ph)	1/0/100	6	>99	14.5	13.2	1.23
10^g^	[**3a**]Al(OCH_2_Ph)	1/0/100	6	67	9.8	8.8	1.16
11^h^	[**3a**]Al(OCH_2_Ph)	1/0/100	6	12	1.8	NA^i^	NA^i^
12^j^	[**3a**]AlMe	1/1/100	48	98	16.1	14.3^k^	1.13
13^j^	[**3a**]AlMe	1/1/200	48	92	28.5	21.1^k^	1.14

a *Unless otherwise noted, all reactions were conducted in toluene (2.24 mL total) at 70°C with benzyl alcohol being the initiator, [cat]_0_ = 8.3 mM*.

b *Determined by ^1^H NMR analysis*.

c *Calculated from {fw of LA × ([LA]_0_/([cat]_0_[I]_0_)) × conversion} + fw of initiator, assuming one propagating chain per aluminum atom*.

d *Measured by GPC in THF, calibrated with polystyrene standards*.

e *Multiplied by a corrected factor of 0.58 (Save et al., 2002)*.

f *Data selected from Chang et al. (2016)*.

g *Reaction run in THF*.

h *Reaction run at room temperature*.

i *Not applicable due to the formation of low Mn oligomers*.

j *Reaction run with MePEG2000 as the initiator*.

k *Corrected by applying a factor of 0.58 to the PLA block*.

**Figure 7:**
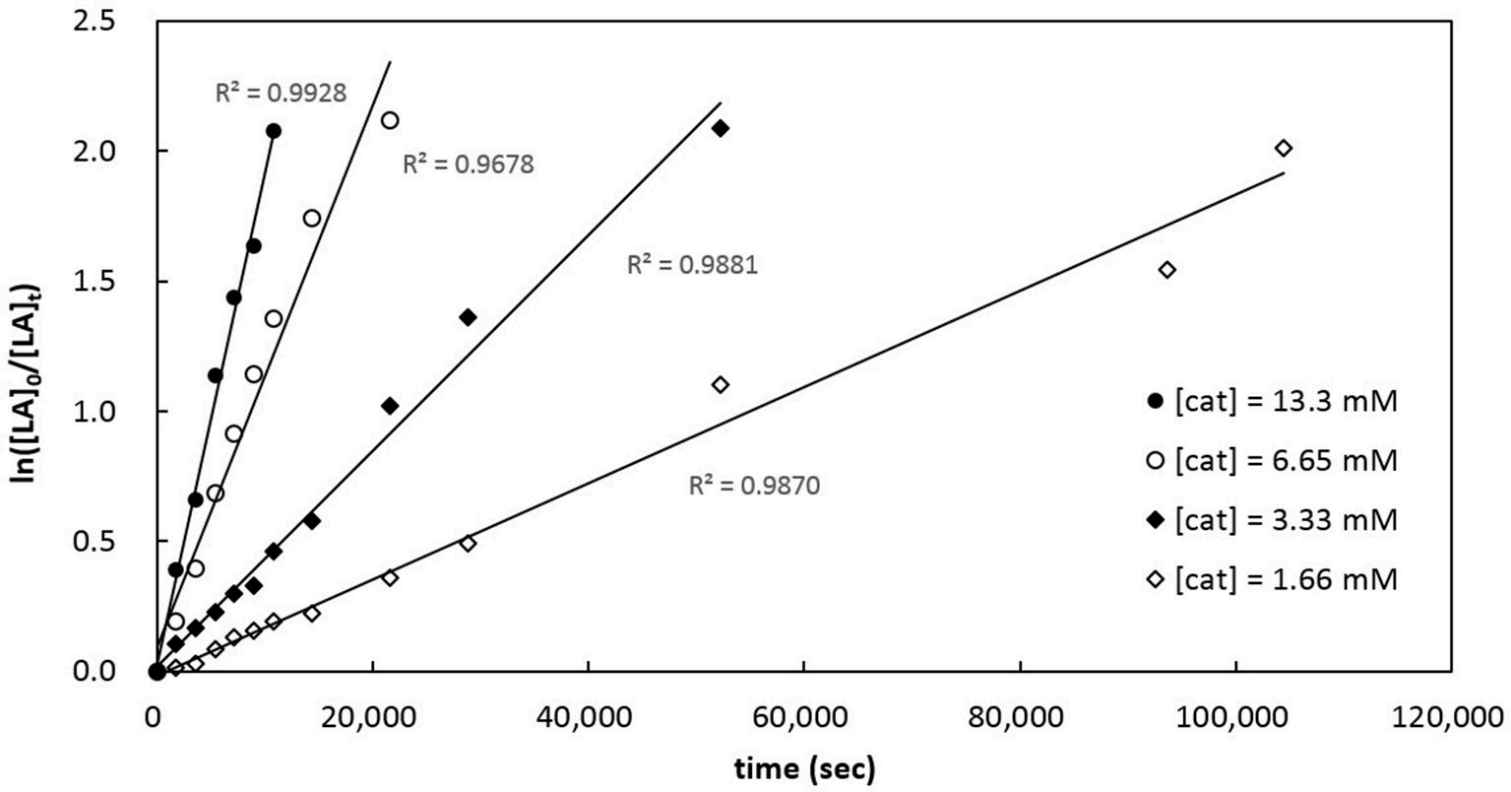
Semilogarithmic plots of *rac*-LA conversion with time employing catalytic [**3a**]Al(OCH_2_Ph) in toluene-*d*_8_ at 70°C. [*rac*-LA]_0_ = 208 mM; i, [cat]_0_ = 13.3 mM, *k*_obs_ = 1.89 (7) × 10^−4^ s^−1^; ii, [cat]_0_ = 6.65 mM, *k*_obs_ = 1.04 (7) × 10^−4^ s^−1^; iii, [cat]_0_ = 3.33 mM, *k*_obs_ = 4.15 (15) × 10^−5^ s^−1^; iv, [cat]_0_ = 1.66 mM, *k*_obs_ = 1.85 (6) × 10^−5^ s^−1^.

**Figure 8:**
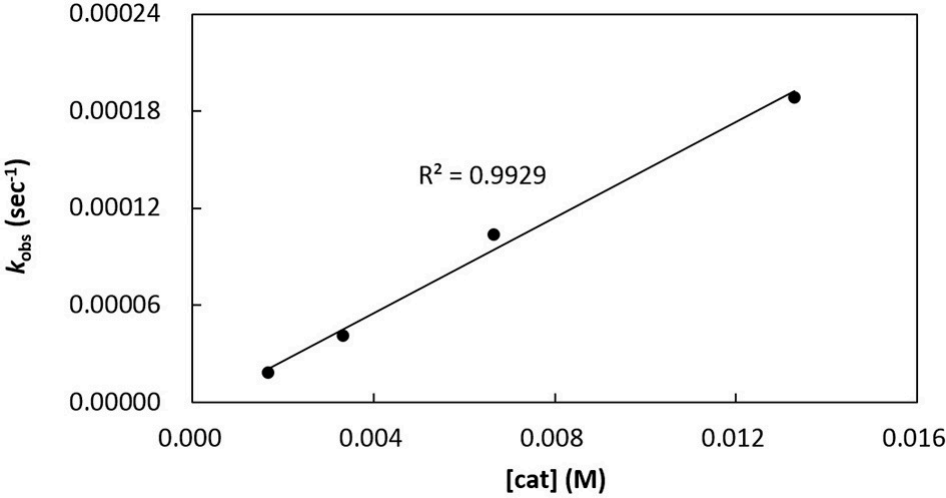
Plot of *k*_obs_ vs. concentrations of [**3a**]Al(OCH_2_Ph) for ROP of *rac*-LA in toluene-*d*_8_ at 70°C; [*rac*-LA]_0_ = 208 mM.

Kinetics of *rac*-LA polymerization by catalytic [**3a**]Al(OCH_2_Ph) was studied. Monitoring the reaction progress by ^1^H NMR spectroscopy reveals linear semilogarithmic plots for *rac*-LA consumptions vs. time (Figure 7), indicating a pseudo-first order dependence of the polymerization rates on the concentrations of *rac*-LA, i.e., –d[*rac*-LA]/dt = *k*_obs_[*rac*-LA]^1^, where *k*_obs_ = *k*_p_[catalyst]^x^ and *k*_p_ = propagation rate constant. A plot of the observed rate constants vs. concentrations of [**3a**]Al(OCH_2_Ph) shows a linear dependence of the former on the latter (Figure 8), thus giving x = 1. The overall rate law of this catalysis is therefore expressed as –d[*rac*-LA]/dt = *k*_p_[catalyst][*rac*-LA], where *k*_p_ = 1.47 (9) × 10^−2^ L mol^−1^ s^−1^ at 70°C.

**Figure 9:**
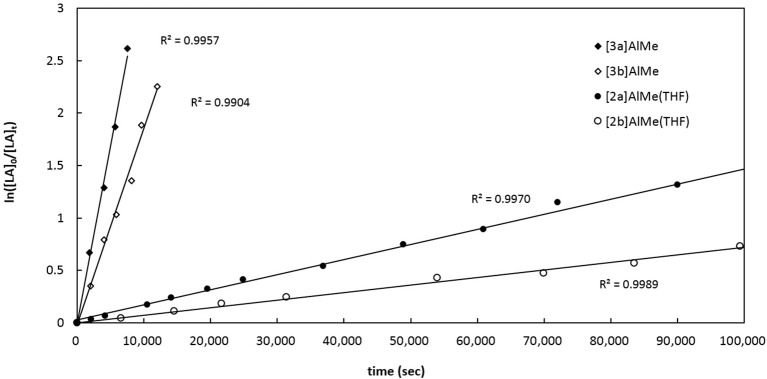
Semilogarithmic plots of *rac*-LA conversion with time employing (i) [**3a**]AlMe, *k*_obs_ = 3.40 (13) × 10^−4^ s^−1^ (ii) [**3b**]AlMe, *k*_obs_ = 1.87 (8) × 10^−4^ s^−1^ (iii) [**2a**]AlMe(THF), *k*_obs_ = 1.44 (2) × 10^−5^ s^−1^ (iv) [**2b**]AlMe(THF), *k*_obs_ = 7.19 (7) × 10^−6^ s^−1^. Conditions: [cat]_0_ = [PhCH_2_OH]_0_ = 6.7 mM, [*rac*-LA] = 670 mM, toluene-*d*_8_, 70°C.

To kinetically quantify the P-substituent effect, we turn our attention to the relative ROP rates of *rac*-LA by catalytic [**3a**]AlMe, [**3b**]AlMe, [**2a**]AlMe(THF), and [**2b**]AlMe(THF) in the presence of one equiv of benzyl alcohol. Figure 9 depicts their semilogarithmic plots of *rac*-LA conversions with time. As a result, the reactivity of these catalyst precursors follows the order of [**3a**]AlMe > [**3b**]AlMe > [**2a**]AlMe(THF) > [**2b**]AlMe(THF). In this catalysis, [**3a**]AlMe is more reactive than [**3b**]AlMe by 1.8 times and [**2a**]AlMe(THF) is more reactive than [**2b**]AlMe(THF) by 2.0 times. More importantly, [**3a**]AlMe is more reactive than [**2a**]AlMe(THF) by 23.6 times and [**3b**]AlMe is more reactive than [**2b**]AlMe(THF) by 26.1 times. Collectively, *tert*-butyl is a superior P-substituent to phenyl and P = O is a superior biphenolate bridge to P in view of offering higher reactivity in this ROP catalysis.

## Conclusions

We have prepared the first examples of biphenolate phosphinoxide complexes of aluminum and characterized their solution and solid state structures by NMR spectroscopy and X-ray crystallography, respectively. The coordination chemistry of these complexes is compared with those of their amine **1** (Liang et al., 2013b) and phosphine **2** (Chang et al., 2016) counterparts, so are their catalytic activities with respect to ROP of ε-CL and *rac*-LA. In addition to the inherent discrepancies of neutral donors in **1**, **2**, and **3**, the 6-membered chelating rings rendered by the rigid **3** are advantageous to enhance substantially the reactivity of aluminum complexes in comparison with those derived from **1** and **2** as demonstrated by their relative ROP rates. Of particular note is also the competence of **3a** complexes in living ROP of ε-CL and *rac*-LA.

## Experimental Section

### General Procedures

Unless otherwise specified, all experiments were performed under nitrogen using standard Schlenk or glovebox techniques. Compounds H_2_[*t*BuP(2-O-3,5-*t*Bu_2_C_6_H_2_)_2_] (H_2_[**2a**]) (Hsu and Liang, 2010) and H_2_[PhP(O)(2-O-3,5-*t*Bu_2_C_6_H_2_)_2_] (H_2_[**3b**]) (Siefert et al., 2000) were prepared according to literature procedures. ε-CL was dried over CaH_2_ (1 wt%) at 80°C for 0.5 h and distilled under reduced pressure. *rac-*LA was purified by recrystallization (four times) from mixtures of toluene and ethyl acetate. All other chemicals were obtained from commercial vendors and used as received. All solvents were reagent grade or better and purified by standard methods. All NMR spectra were recorded at room temperature in specified solvents unless otherwise noted. Chemical shifts (δ) are listed as parts per million downfield from tetramethylsilane and coupling constants (*J*) are listed in hertz. Routine coupling constants are not listed. ^1^H NMR spectra are referenced using the residual solvent peak at δ 7.16 for C_6_D_6_ or δ 2.09 for toluene-*d*_8_ (the most upfield signal). ^13^C NMR spectra are referenced using the internal solvent peak at δ 128.39 for C_6_D_6_. The assignment of the carbon atoms for all new compounds is based on the DEPT ^13^C NMR spectroscopy. ^31^P NMR spectra are referenced externally using 85% H_3_PO_4_ at δ 0. The NOE data were obtained with a ^1^H NMR NOEDIF experiment. GPC analyses were carried out at 45°C with HPLC grade THF supplied at a constant flow rate of 1.0 mL/min. Molecular weights (*M*n and *M*w) were determined by interpolation from calibration plots established with polystyrene standards. Mass spectra were recorded on a Finnigan MAT 95XL Mass Spectrometer. Elemental analysis was performed on a Heraeus CHN-O Rapid analyzer.

### X-Ray Crystallography

Crystallographic data for H_2_[**3a**], [**3a**]AlMe•AlMe_3_, and {[**3a**]Al(μ_2_-OCH_2_Ph)}_2_ are available in Supplementary Material. Data were collected on a diffractometer with graphite monochromated Mo-Kα radiation (λ = 0.7107 Å). Structures were solved by direct methods and refined by full matrix least squares procedures against *F*^2^ using SHELXL-97 (Sheldrick, 1998). All full-weight non-hydrogen atoms were refined anisotropically. Hydrogen atoms were placed in calculated positions. CCDC 1540207, 1540209, 1540210 contain the supplementary crystallographic data for this paper. These data can be obtained free of charge from The Cambridge Crystallographic Data Centre via www.ccdc.cam. ac.uk/data_request/cif.

### Synthesis of H_2_[*t*BuP(O)(2-O-3,5-*t*Bu_2_C_6_H_2_)_2_] (H_2_[3a])

To a THF solution (10 mL) of H_2_[**2a**] (500 mg, 1.0 mmol) was added H_2_O_2_ (0.23 mL, 30% in aqueous solution, 2.0 mmol, 2 equiv) under ambient conditions. The solution was stirred at room temperature for 3 h and evaporated to dryness under reduced pressure. The solid thus obtained was washed with acetonitrile (4 mL) to afford the product as an off-white solid; yield 450 mg (87%). Colorless crystals suitable for X-ray diffraction analysis were grown from a concentrated THF solution at −35°C. ^1^H NMR (C_6_D_6_, 300 MHz) δ 12.19 (s, 2, ArO*H*), 7.62 (s, 2, Ar*H*), 7.55 (d, 2, *J*_HP_ = 12, Ar*H*), 1.52 (s, 18, ArC*Me*_3_), 1.27 (s, 18, ArC*Me*_3_), 1.20 (d, 9, ^3^*J*_HP_ = 15, PC*Me*_3_). ^31^P{^1^H} NMR (C_6_D_6_, 121.5 MHz) δ 65.16. ^13^C{^1^H} NMR (C_6_D_6_, 75 MHz) δ 162.1 (s, *C*), 140.2 (d, *J*_CP_ = 11.3, *C*), 139.1 (d, *J*_CP_ = 6.8, *C*), 129.2 (s, *Ar*H), 125.2 (d, *J*_CP_ = 9.8, *Ar*H), 109.1 (d, *J*_CP_ = 89.3, *C*), 36.9 (d, *J*_CP_ = 67.5, P*C*Me_3_), 35.8 (s, Ar*C*Me_3_), 34.5 (s, Ar*C*Me_3_), 31.8 (s, ArC*Me*_3_), 30.0 (s, ArC*Me*_3_), 24.5 (s, PC*Me*_3_). Anal. Calcd for C_32_H_51_O_3_P: C, 74.66; H, 9.99. Found: C, 74.65; H, 9.94. MS (EI): calcd for C_32_H_51_O_3_P *m*/*z* 514.4, found *m*/*z* 514.5.

### Synthesis of [3a]AlMe

A THF solution (6 mL) of AlMe_3_ (0.20 mL, 2 M in toluene, 0.4 mmol) was chilled to 0°C. To this was added a pre-chilled THF solution (6 mL) of H_2_[**3a**] (206.2 mg, 0.4 mmol) at 0°C. The reaction solution was stirred at room temperature for 1 h and evaporated to dryness under reduced pressure. The solid thus obtained was washed with pentane (2 mL) to afford the product as an off-white solid; yield 215.2 mg (97%). ^1^H NMR (C_6_D_6_, 300 MHz) δ 7.58 (d, 2, *J*_HH_ = 3.0, Ar), 7.41 (dd, 2, *J*_HP_ = 15.0 and *J*_HH_ = 3.0, Ar), 1.54 (s, 18, ArC*Me*_3_), 1.36 (d, 9, ^3^*J*_HP_ = 15, PC*Me*_3_), 1.19 (s, 18, ArC*Me*_3_), 0.01 (s, 3, Al*Me*). ^31^P{^1^H} NMR (C_6_D_6_, 121.5 MHz) δ 61.37. ^13^C{^1^H} NMR (C_6_D_6_, 75 MHz) δ 163.9 (s, *C*), 141.6 (d, *J*_CP_ = 6.8, *C*), 139.1 (d, *J*_CP_ = 12.8, *C*), 129.4 (s, *C*H), 122.5 (d, *J*_CP_ = 12.8, *C*H), 114.1 (d, *J*_CP_ = 90.0, *C*), 35.9 (s, Ar*C*Me_3_), 35.2 (d, *J*_CP_ = 68.3, P*C*Me_3_), 34.3 (s, Ar*C*Me_3_), 31.6 (s, ArC*Me*_3_), 29.7 (s, ArC*Me*_3_), 26.1 (s, PC*Me*_3_), −16.4 (s, Al*Me*). Anal. Calcd for C_33_H_52_AlO_3_P: C, 71.44; H, 9.45. Found: C, 71.37; H, 9.38. MS (EI): calcd for C_33_H_52_AlO_3_P *m*/*z* 554.4, found *m*/*z* 554.5.

### Synthesis of [3b]AlMe

The procedures were all identical to those of [**3a**]AlMe except using H_2_[**3b**] in the place of H_2_[**3a**], affording the product as an off-white solid; yield 95%. ^1^H NMR (C_6_D_6_, 300 MHz) δ 7.70 (m, 2, Ar), 7.64 (d, 2, *J*_HH_ = 1.2, Ar), 7.14 (d, 2, *J*_HH_ = 1.2, Ar), 7.02 (m, 1, Ar), 6.92 (m, 2, Ar), 1.62 (s, 18, ArC*Me*_3_), 1.13 (s, 18, ArC*Me*_3_), 0.05 (s, 3, Al*Me*). ^31^P{^1^H} NMR (C_6_D_6_, 121.5 MHz) δ 55.06. ^13^C{^1^H} NMR (C_6_D_6_, 75 MHz) δ 164.4 (s, *C*), 141.4 (s, *C*), 139.4 (d, *J*_CP_ = 7.5, *C*), 134.1 (d, *J*_CP_ = 6.8, *C*H), 129.8 (s, *C*H), 129.0 (d, *J*_CP_ = 6.8*, C*H), 128.4 (s, *C*H), 125.1 (d, *J*_CP_ = 6.8, *C*H), 112.8 (d, *J*_CP_ = 62.3, *C*), 35.9 (s, *C*Me_3_), 34.2 (s, *C*Me_3_), 31.5 (s, C*Me*_3_), 29.7 (s, C*Me*_3_), −16.5 (s, Al*Me*). Anal. Calcd for C_35_H_48_AlO_3_P: C, 73.13; H, 8.42. Found: C, 72.75; H, 8.26.

### Synthesis of {[3a]Al(μ_2_-OCH_2_Ph)}_2_

A THF solution of [**3a**]AlMe was prepared *in situ* as describe above from the reaction of H_2_[**3a**] (206.2 mg, 0.4 mmol) and AlMe_3_ (0.20 mL, 2 M in toluene, 0.4 mmol). To this was added PhCH_2_OH (43.2 mg, 0.4 mmol). The reaction solution was stirred at room temperature overnight and evaporated to dryness under reduced pressure. The solid thus obtained was washed with pentane (4 mL × 2) to afford the product as an off-white solid; yield 168.4 mg (65%). ^1^H NMR (C_6_D_6_, 300 MHz) δ 7.76 (d, 4, *J*_HH_ = 7.5, Ar), 7.57 (d, 4, *J*_HH_ = 2.1, Ar), 7.38 (dd, 4, *J*_HP_ = 13.5 and *J*_HH_ = 2.1, Ar), 7.21 (t, 4, *J*_HH_ = 7.5, Ar), 7.01 (t, 2, *J*_HH_ = 7.2, Ar), 5.72 (s, 4, OC*H*_2_Ph), 1.54 (s, 36, ArC*Me*_3_), 1.18 (s, 36, ArC*Me*_3_), 1.12 (d, 18, *J*_HP_ = 14.7, PC*Me*_3_). ^31^P{^1^H} NMR (C_6_D_6_, 121.5 MHz) δ 56.74. ^13^C{^1^H} NMR (C_6_D_6_, 75 MHz) δ 164.9 (s, *C*), 143.0 (s, ipso-OCH_2_*Ph*), 141.4 (d, *J*_CP_ = 6.8, *C*), 137.9 (d, *J*_CP_ = 12.9, *C*), 128.8 (s, *C*H), 128.3 (s, *C*H), 125.8 (s, para-OCH_2_*Ph*), 125.6 (s, *C*H), 122.4 (d, *J*_CP_ = 12.6, *C*H), 114.01 (d, *J*_CP_ = 91.5, *C*), 67.5 (s, O*C*H_2_Ph), 35.9 (s, Ar*C*Me_3_), 35.2 (d, *J*_CP_ = 71.2, P*C*Me_3_), 34.2 (s, Ar*C*Me_3_), 31.6 (s, ArC*Me*_3_), 30.1 (s, ArC*Me*_3_), 25.8 (s, PC*Me*_3_). Anal. Calcd for (C_39_H_56_AlO_4_P)_2_: C, 72.40; H, 8.73. Found: C, 72.06; H, 8.47.

### Synthesis of {[3b]Al(μ_2_-OCH_2_Ph)}_2_

The procedures were all identical to those of {[**3a**]Al(μ_2_-OCH_2_Ph)}_2_ except using H_2_[**3b**] in the place of H_2_[**3a**], affording the product as an off-white solid; yield 68%. ^1^H NMR (toluene-*d*_8_, 300 MHz) δ 7.60 (m, 8, Ar), 7.25 (m, 4, Ar), 6.90–7.09 (m, 16, Ar), 5.65 (s, 4, OC*H*_2_Ph), 1.55 (s, 36, ArC*Me*_3_), 1.13 (s, 36, ArC*Me*_3_). ^31^P{^1^H} NMR (toluene-*d*_8_, 121.5 MHz) δ 51.75. ^13^C{^1^H} NMR (C_6_D_6_, 75 MHz) δ 165.5 (s, *C*), 143.4 (s, ipso-OCH_2_*Ph*), 141.5 (d, *J*_CP_ = 7.7, *C*), 138.2 (d, *J*_CP_ = 13.4, *C*), 133.9 (d, *J*_CP_ = 10.4, *C*H), 132.9 (s, *C*H), 129.2 (s, *C*H), 128.4 (s, *C*H), 127.6 (s, *C*H), 126.0 (s, *C*H), 125.2 (d, *J*_CP_ = 14.3, *C*H), 125.0 (d, *J*_CP_ = 12.2, *C*H), 112.9 (d, *J*_CP_ = 104.1, *C*), 67.7 (s, O*C*H_2_Ph), 35.9 (s, Ar*C*Me_3_), 34.1 (s, Ar*C*Me_3_), 31.5 (s, ArC*Me*_3_), 30.2 (s, ArC*Me*_3_). Anal. Calcd for (C_41_H_52_AlO_4_P)_2_: C, 73.85; H, 7.86. Found: C, 73.51; H, 7.65.

### Catalytic ROP of ε-CL or *rac*-LA (Tables 1, 2)

A toluene solution (1 mL) containing an alcohol initiator (PhCH_2_OH or MePEG2000) where appropriate and monomer (ε-CL or *rac*-LA having prescribed [monomer]_0_/[catalyst]_0_ ratios) was heated in an oil bath at 70°C. To this was added a toluene solution (1.24 mL) of catalyst [**3a-b**]AlMe (0.0187 mmol) or {[**3a-b**]Al(μ_2_-OCH_2_Ph)}_2_ (0.00935 mmol). The reaction solution was stirred at 70°C for a period of prescribed time and quenched with a methanol solution of HCl. The solid thus precipitated was washed with hexane, isolated, and dried under reduced pressure until constant weights.

### Kinetic Studies on ROP of *rac*-LA

The procedures were similar to those described above except that the reactions were conducted in toluene-*d*_8_. The monomer conversion was monitored over time by ^1^H NMR spectrometry.

## Author Contributions

All authors made substantial contributions to this work. X-RZ and Y-NC conducted experiments, analyzed results, and tabulated data. K-WH participated in the development and discussion of this work. L-CL conceived the project, directed the investigations, and composed the manuscript.

### Conflict of Interest Statement

The authors declare that the research was conducted in the absence of any commercial or financial relationships that could be construed as a potential conflict of interest.

## Supplementary Material

X-ray crystallographic data for H_2_[**3a**], [**3a**]AlMe•AlMe_3_, and {[**3a**]Al(μ_2_-OCH_2_Ph)}_2_.

## References

[B1] Alcazar-Roman L. M. O'Keefe B. J. Hillmyer M. A. Tolman W. B. (2003). Electronic influence of ligand substituents on the rate of polymerization of epsilon-caprolactone by single-site aluminium alkoxide catalysts. Dalton Trans. 33, 3082–3087. 10.1039/B303760F

[B2] Buffet J. C. Martin A. N. Kol M. Okuda J. (2011). Controlled stereoselective polymerization of lactide monomers by group 4 metal initiators that contain an (OSSO)-type tetradentate bis(phenolate) ligand. Polym. Chem. 2, 2378–2384. 10.1039/c1py00266j

[B3] Buffet J. C. Okuda J. (2011). Group 4 metal initiators for the controlled stereoselective polymerization of lactide monomers. Chem. Commun. 47, 4796–4798. 10.1039/c1cc10149h 21409212

[B4] Chang Y.-N. Lee P.-Y. Zou X.-R. Huang H.-F. Chen Y.-W. Liang L.-C. (2016). Aluminum complexes containing biphenolate phosphine ligands: synthesis and living ring-opening polymerization catalysis. Dalton Trans. 45, 15951–15962. 10.1039/C6DT02143C 27406437

[B5] Chang Y.-N. Liang L.-C. (2007). Preparation and structural characterization of group 1 metal complexes containing a chelating biphenolato phosphine ligand. Inorg. Chim. Acta 360, 136–142. 10.1016/j.ica.2006.07.050

[B6] Chen H. L. Dutta S. Huang P. Y. Lin C. C. (2012). Preparation and characterization of aluminum alkoxides coordinated on salen-type ligands: highly stereoselective ring-opening polymerization of rac-lactide. Organometallics 31, 2016–2025. 10.1021/om201281w

[B7] Chmura A. J. Davidson M. G. Jones M. D. Lunn M. D. Mahon M. F. (2006). Group 4 complexes of amine bis(phenolate)s and their application for the ring opening polymerisation of cyclic esters. Dalton Trans. 2006, 887–889. 10.1039/B513345A 16462948

[B8] Fryzuk M. D. Giesbrecht G. R. Olovsson G. Rettig S. J. (1996). Synthesis and characterization of four- and five-coordinate organoaluminum complexes incorporating the amido diphosphine ligand system N(SiMe2CH2PiPr2)2. Organometallics 15, 4832–4841. 10.1021/om9604583

[B9] Fryzuk M. D. Giesbrecht G. R. Rettig S. J. (1998). Pyramidal inversion at phosphorus facilitated by the presence of proximate Lewis acids. Coordination chemistry of group 13 elements with the macrocyclic bis(amidophosphine) ligand P2N2 (P2N2 = PhP(CH2SiMe2NSiMe2CH2)2PPh). Inorg. Chem. 37, 6928–6934. 10.1021/ic9805978 11670831

[B10] Gao B. Li D. N. Li Y. H. Duan Q. Duan R. L. Pang X. (2015). Ring-opening polymerization of lactide using chiral salen aluminum complexes as initiators: high productivity and stereoselectivity. N. J. Chem. 39, 4670–4675. 10.1039/C5NJ00469A

[B11] Gendler S. Segal S. Goldberg I. Goldschmidt Z. Kol M. (2006). Titanium and zirconium complexes of dianionic and trianionic amine - Phenolate-type ligands in catalysis of lactide polymerization. Inorg. Chem. 45, 4783–4790. 10.1021/ic052120j 16749843

[B12] He L.-P. Liu J.-Y. Pan L. Li Y.-S. (2008). Ethylene polymerization of the new titanium complexes bearing a phosphine oxide-bridged bisphenolato ligand. J. Polym. Sci. A 46, 7062–7073. 10.1002/pola.23012

[B13] Hillmyer M. A. Tolman W. B. (2014). Aliphatic polyester block polymers: renewable, degradable, and sustainable. Acc. Chem. Res. 47, 2390–2396. 10.1021/ar500121d 24852135

[B14] Hormnirun P. Marshall E. L. Gibson V. C. Pugh R. I. White A. J. (2006). Study of ligand substituent effects on the rate and stereoselectivity of lactide polymerization using aluminum salen-type initiators. Proc. Natl. Acad. Sci. U.S.A. 103, 15343–15348. 10.1073/pnas.0602765103 17032771 PMC1622826

[B15] Hsu Y.-L. Liang L.-C. (2010). Alkali metal complexes of a tert-butylphosphine-bridged biphenolate ligand. Organometallics 29, 6201–6208. 10.1021/om100495e 21428317

[B16] Huang M.-H. Liang L.-C. (2004). Amido pincer complexes of palladium: synthesis, structure, and catalytic Heck reaction. Organometallics 23, 2813–2816. 10.1021/om049888g

[B17] Huang Y. Wang W. Lin C. C. Blake M. P. Clark L. Schwarz A. D.. (2013). Potassium, zinc, and magnesium complexes of a bulky OOO-tridentate bis(phenolate) ligand: synthesis, structures, and studies of cyclic ester polymerisation. Dalton Trans. 42, 9313–9324. 10.1039/c3dt50135c 23435514

[B18] Hung Y.-T. Chen M.-T. Huang M.-H. Kao T.-Y. Liu Y.-S. Liang L.-C. (2014). Catalytic Sonogashira couplings mediated by an amido pincer complex of palladium. Inorg. Chem. Front. 1, 405–413. 10.1039/c3qi00086a

[B19] Jones M. D. Brady L. McKeown P. Buchard A. Schäfer P. M. Thomas L. H.. (2015). Metal influence on the iso- and hetero-selectivity of complexes of bipyrrolidine derived salan ligands for the polymerisation of rac-lactide. Chem. Sci. 6, 5034–5039. 10.1039/C5SC01819F 29142728 PMC5664169

[B20] Kamber N. E. Jeong W. Waymouth R. M. Pratt R. C. Lohmeijer B. G. Hedrick J. L. (2007). Organocatalytic ring-opening polymerization. Chem. Rev. 107, 5813–5840. 10.1021/cr068415b 17988157

[B21] Kirk S. M. Kociok-Kohn G. Jones M. D. (2016). Zirconium vs aluminum salalen initiators for the production of biopolymers. Organometallics 35, 3837–3843. 10.1021/acs.organomet.6b00718

[B22] Klitzke J. S. Roisnel T. Kirillov E. Casagrande O. Carpentier J.-F. (2014a). Discrete O-Lactate and β-alkoxybutyrate aluminum pyridine–bis(naphtholate) complexes: models for mechanistic investigations in the ring-opening polymerization of lactides and β-lactones. Organometallics 33, 5693–5707. 10.1021/om401214q

[B23] Klitzke J. S. Roisnel T. Kirillov E. Casagrande O. Carpentier J.-F. (2014b). Yttrium– and aluminum–bis(phenolate)pyridine complexes: catalysts and model compounds of the intermediates for the stereoselective ring-opening polymerization of racemic lactide and β-butyrolactone. Organometallics 33, 309–321. 10.1021/om401047r

[B24] Lee C.-L. Lin Y.-F. Jiang M.-T. Lu W.-Y. Vandavasi J. K. Wang L.-F. (2017). Improvement in aluminum complexes bearing schiff bases in ring-opening polymerization of ε-caprolactone: a five-membered-ring system. Organometallics 36, 1936–1945. 10.1021/acs.organomet.7b00068

[B25] Lee P.-Y. Liang L.-C. (2009). Synthesis and structural characterization of five-coordinate aluminum complexes containing diarylamido diphosphine ligands. Inorg. Chem. 48, 5480–5487. 10.1021/ic802030d 19400561

[B26] Lee W.-Y. Liang L.-C. (2005). Organoaluminium complexes incorporating an amido phosphine chelate with a pendant amine arm. Dalton Trans. 1952–1956. 10.1039/b502873f 15909042

[B27] Liang L.-C. (2006). Metal complexes of chelating diarylamido phosphine ligands. Coord. Chem. Rev. 250, 1152–1177. 10.1016/j.ccr.2006.01.001

[B28] Liang L.-C. Chen F.-Y. Huang M.-H. Cheng L.-C. Li C.-W. Lee H. M. (2010). Aluminium complexes of bidentate N,O- and N,N-ligands derived from oxidative functionalization of amido phosphines: synthesis, structure and reactivity. Dalton Trans. 39, 9941–9951. 10.1039/c0dt00418a 20838684

[B29] Liang L.-C. Chien C.-C. Chen M.-T. Lin S.-T. (2013a). Zirconium and hafnium complexes containing N-alkyl-substituted amine biphenolate ligands: unexpected ligand degradation and divergent complex constitutions governed by N-alkyls. Inorg. Chem. 52, 7709–7716. 10.1021/ic400891b 23773167

[B30] Liang L.-C. Chien P.-S. Huang M.-H. (2005a). Catalytic suzuki coupling reactions by amido phosphine complexes of palladium. Organometallics 24, 353–357. 10.1021/om0492395

[B31] Liang L.-C. Chien P.-S. Huang Y.-L. (2006). Intermolecular arene C-H activation by nickel(II). J. Am. Chem. Soc. 128, 15562–15563. 10.1021/ja065505p 17147345

[B32] Liang L.-C. Hsu Y.-L. Lin S.-T. (2011). Group 4 complexes of a tert-butylphosphine-bridged biphenolate ligand. Inorg. Chem. 50, 3363–3372. 10.1021/ic102194z 21428317

[B33] Liang L.-C. Huang M.-H. Hung C.-H. (2004). Aluminum complexes incorporating bidentate amido phosphine ligands. Inorg. Chem. 43, 2166–2174. 10.1021/ic035373q 15018541

[B34] Liang L.-C. Lee W.-Y. Hung C.-H. (2003a). Amido phosphine complexes of zinc. Inorg. Chem. 42, 5471–5473. 10.1021/ic0346228 12950189

[B35] Liang L.-C. Lin J.-M. Hung C.-H. (2003b). Nickel(II) complexes of bis(2-diphenylphosphinophenyl)amide. Organometallics 22, 3007–3009. 10.1021/om030237e

[B36] Liang L.-C. Lin J.-M. Lee W.-Y. (2005b). Benzene C-H activation by platinum(II) complexes of bis(2-diphenylphosphinophenyl)amide. Chem. Commun. 2462–2464. 10.1039/B501520K 15886771

[B37] Liang L.-C. Lin S.-T. Chien C.-C. (2013b). Aluminum complexes of tridentate amine biphenolate ligands containing distinct N-alkyls: synthesis and catalytic ring-opening polymerization. J. Chin. Chem. Soc. 60, 710–718. 10.1002/jccs.201200559

[B38] Liang L.-C. Lin S.-T. Chien C.-C. (2013c). Lithium complexes of tridentate amine biphenolate ligands containing distinct N-alkyl substituents. Polyhedron 52, 1090–1095. 10.1016/j.poly.2012.06.069

[B39] Liang L.-C. Lin S.-T. Chien C.-C. (2013d). Titanium complexes of tridentate aminebiphenolate ligands containing distinct N-alkyls: profound N-substituent effect on ring-opening polymerization catalysis. Inorg. Chem. 52, 1780–1786. 10.1021/ic301551v 23362792

[B40] Liang L.-C. Lin S.-T. Chien C.-C. Chen M.-T. (2013e). Zirconium and hafnium complexes containing N-alkyl substituted amine biphenolate ligands: coordination chemistry and living ring-opening polymerization catalysis. Dalton Trans. 42, 9286–9293. 10.1039/C3DT50152C 23518868

[B41] MacDonald J. P. Sidera M. Fletcher S. P. Shaver M. P. (2016). Living and immortal polymerization of seven and six membered lactones to high molecular weights with aluminum salen and salan catalysts. Eur. Polym. J. 74, 287–295. 10.1016/j.eurpolymj.2015.11.032

[B42] MacLachlan E. A. Fryzuk M. D. (2005). A new arene-bridged diamidophosphine ligand and its coordination chemistry with zirconium(IV). Organometallics 24, 1112–1118. 10.1021/om049165x

[B43] MacLachlan E. A. Hess F. M. Patrick B. O. Fryzuk M. D. (2007). New side-on bound dinitrogen complexes of zirconium supported by an arene-bridged diamidophosphine ligand and their reactivity with dihydrogen. J. Am. Chem. Soc. 129, 10895–10905. 10.1021/ja073753v 17685617

[B44] McKeown P. Davidson M. G. Kociok-Köhn G. Jones M. D. (2016). Aluminium salalens vs. salans: “Initiator Design” for the isoselective polymerisation of rac-lactide. Chem. Commun. 52, 10431–10434. 10.1039/C6CC05795K 27487791

[B45] Ovitt T. M. Coates G. W. (2000). Stereoselective ring-opening polymerization of rac-lactide with a single-site, racemic aluminum alkoxide catalyst: synthesis of stereoblock poly(lactic acid). J. Polym. Sci. A 38, 4686–4692. 10.1002/1099-0518(200012)38:1+<4686::AID-POLA80>3.0.CO;2-0

[B46] Paine T. K. Weyhermüller T. Slep L. D. Neese F. Bill E. Bothe E.. (2004). Nonoxovanadium(IV) and oxovanadium(V) complexes with mixed O, X, O-donor ligands (X = S, Se, P., or PO). Inorg. Chem. 43, 7324–7338. 10.1021/ic040052f 15530082

[B47] Pang X. Duan R. L. Li X. Hu C. Y. Wang X. H. Chen X. S. (2018). Breaking the paradox between catalytic activity and stereoselectivity: rac-lactide polymerization by trinuclear salen-Al complexes. Macromolecules 51, 906–913. 10.1021/acs.macromol.7b02662

[B48] Phomphrai K. Chumsaeng P. Sangtrirutnugul P. Kongsaeree P. Pohmakotr M. (2010). Reverse orders of reactivities in the polymerization of cyclic esters using N2O2 aluminium alkoxide complexes. Dalton Trans. 39, 1865–1871. 10.1039/B919340E 20449433

[B49] Robert C. Schmid T. E. Richard V. Haquette P. Raman S. K. Rager M. N.. (2017). Mechanistic aspects of the polymerization of lactide using a highly efficient aluminum(III) catalytic system. J. Am. Chem. Soc. 139, 6217–6225. 10.1021/jacs.7b01749 28398052

[B50] Sarazin Y. Carpentier J. F. (2015). Discrete cationic complexes for ring-opening polymerization catalysis of cyclic esters and epoxides. Chem. Rev. 115, 3564–3614. 10.1021/acs.chemrev.5b00033 25897976

[B51] Save M. Schappacher M. Soum A. (2002). Controlled ring-opening polymerization of lactones and lactides initiated by lanthanum isopropoxide, 1. general aspects and kinetics. Macromol. Chem. Phys. 203, 889–899. 10.1002/1521-3935(20020401)203:5/6<889::AID-MACP889>3.0.CO;2-O

[B52] Sheldrick G. M. (1998). SHELXTL, Version 5.1. Madison, WI: Bruker AXA Inc.

[B53] Siefert R. Weyhermuller T. Chaudhuri P. (2000). Isolation, structural and spectroscopic investigations of complexes with tridentate [OPO] and {OOO} donor ligands. J. Chem. Soc. Dalton Trans. 4656–4663. 10.1039/B005693F

[B54] Stopper A. Okuda J. Kol M. (2012). Ring-opening polymerization of lactide with Zr complexes of {ONSO} ligands: from heterotactically inclined to isotactically inclined poly(lactic acid). Macromolecules 45, 698–704. 10.1021/ma2023364

[B55] Su W.-J. Liang L.-C. (2018). Elusive scorpionates: C3-symmetric, formally dianionic, facially tridentate ligands. Inorg. Chem. 57, 553–556. 10.1021/acs.inorgchem.7b02884 29280615

[B56] Tang Z. H. Gibson V. C. (2007). rac-Lactide polymerization using aluminum complexes bearing tetradentate phenoxy-amine ligands. Eur. Polym. J. 43, 150–155. 10.1016/j.eurpolymj.2006.09.023

[B57] Taniyama N. Ohki Y. Tatsumi K. (2014). Synthesis of V/Fe/S clusters using vanadium(III) thiolate complexes bearing a phenoxide-based tridentate ligand. Inorg. Chem. 53, 5438–5446. 10.1021/ic4030603 24840665

[B58] Tanke R. S. Holt E. M. Crabtree R. H. (1991). Ruthenium in an O-donor environment - properties and reactions of ETA-3-(RPO(C6H4O)2)2-, ETA-3-(CPCO(PO(OET)2)3)- and ETA-3-HC(POPH2)3 complexes of ruthenium. Inorg. Chem. 30, 1714–1719. 10.1021/ic00008a009

[B59] Thomas C. M. (2010). Stereocontrolled ring-opening polymerization of cyclic esters: synthesis of new polyester microstructures. Chem. Soc. Rev. 39, 165–173. 10.1039/B810065A 20023847

[B60] Wichmann O. Sillanpaa R. Lehtonen A. (2012). Structural properties and applications of multidentate O,N,O,X ' aminobisphenolate metal complexes. Coord. Chem. Rev. 256, 371–392. 10.1016/j.ccr.2011.09.007

[B61] Zelikoff A. L. Kopilov J. Goldberg I. Coates G. W. Kol M. (2009). New facets of an old ligand: titanium and zirconium complexes of phenylenediamine bis(phenolate) in lactide polymerisation catalysis. Chem. Commun. 6804–6806. 10.1039/B915211C 19885485

[B62] Zhang S.-W. Zhang G.-B. Lu L.-P. Li Y.-S. (2013). Novel vanadium(III) complexes with tridentate phenoxy-phosphine [O,P(= O),O] ligands: synthesis, characterization, and catalytic behavior of ethylene polymerization and copolymerization with 10-undecen-1-ol. J. Polym. Sci. A 51, 844–854. 10.1002/pola.26441

[B63] Zhong Z. Y. Dijkstra P. J. Feijen J. (2002). (salen)Al -mediated, controlled and stereoselective ring-opening polymerization of lactide in solution and without solvent: synthesis of highly isotactic polylactide stereocopolymers from racemic D,L-lactide. Angew. Chem. Int. Ed. 41, 4510–4513. 10.1002/1521-3773(20021202)41:23<4510::AID-ANIE4510>3.0.CO;2-L 12458522

