# Supplementary material for: Enhanced Reactivity of Aluminum Complexes Containing P-Bridged Biphenolate Ligands in Ring-Opening Polymerization Catalysis
Source: Front Chem. 2018 Dec 13;6:607. doi: 10.3389/fchem.2018.00607 (PMC6300491; doi:10.3389/fchem.2018.00607)
Supplement: Supplementary file 1 [file Data_Sheet_1.PDF]

Supplementary Material

for

Enhanced Reactivity of Aluminum Complexes Containing P-bridged Biphenolate Ligands in Ring-opening Polymerization Catalysis

Xue-Ru Zou,[a] Yu-Ning Chang,[a] Kuo-Wei Huang,*[b] and Lan-Chang Liang*[a,c]

[a] Department of Chemistry, National Sun Yat-sen University, Kaohsiung 80424, Taiwan; E-mail: lcliang@mail.nsysu.edu.tw
[b] KAUST Catalysis Center and Division of Physical Sciences and Engineering, King Abdullah University of Science and Technology, Thuwal 23955-6900, Saudi Arabia; E-mail: hkw@kaust.edu.sa
[c] Department of Medicinal and Applied Chemistry, Kaohsiung Medical University, Kaohsiung 80708, Taiwan

Contents

- **Figure S1.** Molecular structure of H_2[**3a**] with thermal ellipsoids drawn at the 35% probability level. The dash lines highlight intramolecular hydrogen bonding between phosphinoxide and hydroxy groups.
- **Table S1.** Crystal data and structure refinement for H_2[**3a**].
- **Table S2.** Crystal data and structure refinement for [**3a**]AlMe•$AlMe_3$.
- **Table S3.** Crystal data and structure refinement for {[**3a**]Al(μ_2-OCH_2Ph)}$_2$.

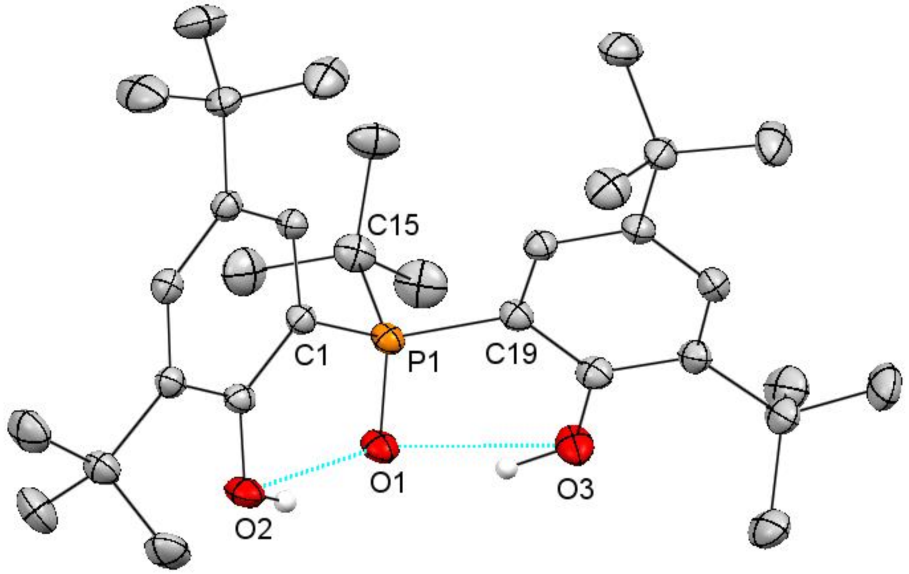

Figure S1. Molecular structure of H_2[**3a**] with thermal ellipsoids drawn at the 35% probability level. All hydrogen atoms are omitted for clarity except those in hydroxy groups. The dash lines highlight intramolecular hydrogen bonding between phosphinoxide and hydroxy groups. Selected bond distances (Å) and angles (deg): C(1)-P(1) 1.7989(19), C(15)-P(1) 1.839(2), C(19)-P(1) 1.8101(19), O(1)-P(1) 1.5170(14), O(1)-P(1)-C(1) 109.29(8), O(1)-P(1)-C(19) 108.46(8), C(1)-P(1)-C(19) 111.57(9), O(1)-P(1)-C(15) 109.10(9), C(1)-P(1)-C(15) 109.03(9), C(19)-P(1)-C(15) 109.36(9).

Table S1. Crystal data and structure refinement for H_2[**3a**].

Empirical formula	C32 H51 O3 P	
Formula weight	514.70	
Temperature	200(2) K	
Wavelength	0.71073 Å	
Crystal system	Monoclinic	
Space group	P 21/n	
Unit cell dimensions	a = 10.0016(4) Å	α= 90°.
	b = 12.0817(5) Å	β= 94.712(3)°.
	c = 26.1347(15) Å	γ = 90°.
Volume	3147.3(3) $Å^3$	
Z	4	
Density (calculated)	1.086 Mg/m^3	
Absorption coefficient	0.115 mm^{-1}	
F(000)	1128	
Crystal size	0.67 x 0.31 x 0.12 mm^3	
Theta range for data collection	1.56 to 25.10°.	
Index ranges	-11<=h<=11, -14<=k<=13, -30<=l<=31	
Reflections collected	19902	
Independent reflections	5568 [R(int) = 0.0386]	
Completeness to theta = 25.10°	99.4 %	
Absorption correction	multi-scan	
Max. and min. transmission	0.9863 and 0.9267	
Refinement method	Full-matrix least-squares on F^2	
Data / restraints / parameters	5568 / 0 / 325	
Goodness-of-fit on F^2	1.105	
Final R indices [I>2sigma(I)]	R1 = 0.0461, wR2 = 0.1321	
R indices (all data)	R1 = 0.0632, wR2 = 0.1515	
Largest diff. peak and hole	0.360 and -0.493 e.$Å^{-3}$	

Table S2. Crystal data and structure refinement for [**3a**]AlMe•$AlMe_3$.

Empirical formula	C36 H61 Al2 O3 P	
Formula weight	626.78	
Temperature	200(2) K	
Wavelength	0.71073 Å	
Crystal system	Triclinic	
Space group	P-1	
Unit cell dimensions	a = 10.7713(9) Å	α= 97.374(3)°.
	b = 13.3342(12) Å	β= 106.713(3)°.
	c = 14.4690(13) Å	γ = 100.970(3)°.
Volume	1916.6(3) $Å^3$	
Z	2	
Density (calculated)	1.086 Mg/m^3	
Absorption coefficient	0.148 mm^{-1}	
F(000)	684	
Crystal size	0.37 x 0.32 x 0.04 mm^3	
Theta range for data collection	2.03 to 25.05°.	
Index ranges	-12<=h<=12, -15<=k<=15, -8<=l<=17	
Reflections collected	15087	
Independent reflections	6547 [R(int) = 0.0792]	
Completeness to theta = 25.05°	96.5 %	
Absorption correction	Semi-empirical from equivalents	
Max. and min. transmission	0.9941 and 0.9473	
Refinement method	Full-matrix least-squares on F^2	
Data / restraints / parameters	6547 / 745 / 382	
Goodness-of-fit on F^2	1.046	
Final R indices [I>2sigma(I)]	R1 = 0.0787, wR2 = 0.1870	
R indices (all data)	R1 = 0.1475, wR2 = 0.2252	
Largest diff. peak and hole	0.591 and -0.684 e.$Å^{-3}$	

Table S3. Crystal data and structure refinement for {[**3a**]Al(μ_2-OCH_2Ph)}$_2$.

Empirical formula	C78 H112 Al2 O8 P2	
Formula weight	1293.58	
Temperature	150(2) K	
Wavelength	0.71073 Å	
Crystal system	Monoclinic	
Space group	P2(1)/c	
Unit cell dimensions	a = 14.8968(8) Å	α= 90°.
	b = 11.1523(7) Å	β= 91.061(5)°.
	c = 23.0975(12) Å	γ = 90°.
Volume	3836.6(4) Å^3	
Z	2	
Density (calculated)	1.120 Mg/m^3	
Absorption coefficient	0.131 mm^{-1}	
F(000)	1400	
Crystal size	0.30 x 0.20 x 0.20 mm^3	
Theta range for data collection	2.90 to 29.24°.	
Index ranges	-20<=h<=14, -15<=k<=14, -31<=l<=31	
Reflections collected	18295	
Independent reflections	8901 [R(int) = 0.0494]	
Completeness to theta = 25.00°	99.8 %	
Absorption correction	Semi-empirical from equivalents	
Max. and min. transmission	0.9744 and 0.9619	
Refinement method	Full-matrix least-squares on F^2	
Data / restraints / parameters	8901 / 0 / 428	
Goodness-of-fit on F^2	1.049	
Final R indices [I>2sigma(I)]	R1 = 0.0657, wR2 = 0.1528	
R indices (all data)	R1 = 0.0951, wR2 = 0.1745	
Largest diff. peak and hole	0.612 and -0.373 e.Å^{-3}	
